# COVID-19-Induced Mesenteric Thrombosis

**DOI:** 10.7759/cureus.12953

**Published:** 2021-01-28

**Authors:** Muhammad Hanif, Zeeshan Ahmad, Abdul Wali Khan, Sidra Naz, FNU Sundas

**Affiliations:** 1 Internal Medicine, Khyber Medical College, Hayatabad Medical Complex Peshawar, Peshawar, PAK; 2 Internal Medicine, Khyber Teaching Hospital, Peshawar, PAK; 3 Internal Medicine, College of Physicians and Surgeons Pakistan, Peshawar, PAK; 4 Internal Medicine, Hayatabad Medical Complex Peshawar, Peshawar, PAK; 5 Internal Medicine, University of Health Sciences (UHS), Lahore, PAK; 6 Internal Medicine, Khyber Medical College, Peshawar, PAK

**Keywords:** covid-19, sars-cov-2, mesenteric thrombosis, thrombophilia screen

## Abstract

Gastrointestinal symptoms, such as diarrhea (most common among gastrointestinal symptoms), nausea/vomiting, anorexia, abdominal pain, abnormal liver enzymes, and pancreatitis, are being increasingly recognized in patients with coronavirus disease 2019 (COVID-19). Moreover, COVID-19 has also been implicated in coagulopathy, especially in patients with severe disease. Here, we report a case of acute intestinal ischemia secondary to superior mesenteric thrombosis in a young female patient with mild COVID-19.

## Introduction

Coronavirus disease 2019 (COVID-19), caused by severe acute respiratory syndrome virus 2 (SARS-CoV-2) - an enveloped, positive-sense, single-stranded RNA belonging to the Coronaviridae family, has been declared a global pandemic by the World Health Organization (WHO) [1]. Since the report of the first case of COVID-19 as a novel type of pneumonia in Wuhan, China, in December 2019, its exponential spread has resulted in almost 19.4 million cases with 0.7 million deaths worldwide [2].

Besides the characteristic presenting symptoms of fever, dry cough, shortness of breath, fatigue, myalgia, and headache; gastrointestinal symptoms, such as diarrhea (most common among gastrointestinal symptoms), nausea/vomiting, anorexia, abdominal pain, abnormal liver enzymes, and pancreatitis, are also being increasingly recognized in patients with COVID-19 [3]. Additionally, COVID-19 has likewise been implicated in coagulopathy, generally in patients with serious ailments. Not only the autopsy findings of venous thromboembolism in COVID-19 cases but also the laboratory findings of increased D-dimer/fibrinogen levels and prolonged prothrombin time seen in patients with severe disease underpin the casual association of COVID-19 and coagulopathy [4]. Likewise, arterial thrombosis, though less common in comparison with venous thrombosis, has also been documented in patients with COVID-19 [5]. Arterial thrombotic complications include stroke, acute limb ischemia, renal infarcts, and, rarely, mesenteric ischemia [5].

Here, we report a case of acute intestinal ischemia secondary to superior mesenteric thrombosis in a young female patient with mild COVID-19 to underscore the importance of monitoring for thrombotic complications via serial measurement of D-dimer, C-reactive protein (CRP) levels, platelet counts, and coagulation panel, even in patients without severe COVID-19. Additionally, such a rare manifestation should be kept in consideration in these pandemic times, and we also recommend the prophylactic use of anticoagulants in patients with COVID-19 if no contraindication exists to prevent these atypical yet fatal thrombotic complications.

## Case presentation

A 20-year-old female presented to the emergency department (Corona desk) with fever and cough for one day. On examination, she was alert, oriented, and conscious, with a temperature of 100.5 F, pulse 72 per minute, and oxygen saturation of 96%. Reverse transcriptase-polymerase chain reaction (RT-PCR) for SARS-CoV-2 nucleic acid done on her nasopharyngeal swab turned out positive. She did not have any comorbidity and was doing well. She was discharged on antipyretic (paracetamol) with the advice to keep herself hydrated and to be self-quarantined. After a week of the first visit, she again presented to the emergency department with a one-day history of abdominal pain and abdominal distension. On examination, she was drowsy and had signs of dehydration with a pulse of regular 92 beats per minute and blood pressure of 80/50 mmHg. Oxygen saturation was 93% at room air with a breathing rate of 22 breaths per minute. The abdomen was tender to palpation in all quadrants. Her blood glucose level was 104 mg/dL and she had decreased urine output. She was given intravenous (IV) fluid, antibiotics, and painkillers, and baseline investigations were sent (Table 1).

**Table 1:** Laboratory findings

Test	Result
Hemoglobin	12.1 g/dL
Total lymphocyte count	15.9 (x10^9^/l)
Red blood cell (RBC)	4.6 (x10^12^/l)
Platelets	633x10^9^/l
Prothrombin time	18 seconds (12 seconds control)
Activated partial thromboplastin time	35 seconds (28 seconds control)
D-dimer	2340 ng/FEUmL (reference value: up to 500 ng/FEUmL)
C-reactive protein	62 mg/dl
Lactate dehydrogenase (LDH)	825 U/L
Serum ferritin level	1435.3 µg/L
Blood urea	49 mg/dL
Creatinine	1 mg/dL
Sodium	147 mEq/l
Potassium	4.8 mEq/l

**Figure 1:**
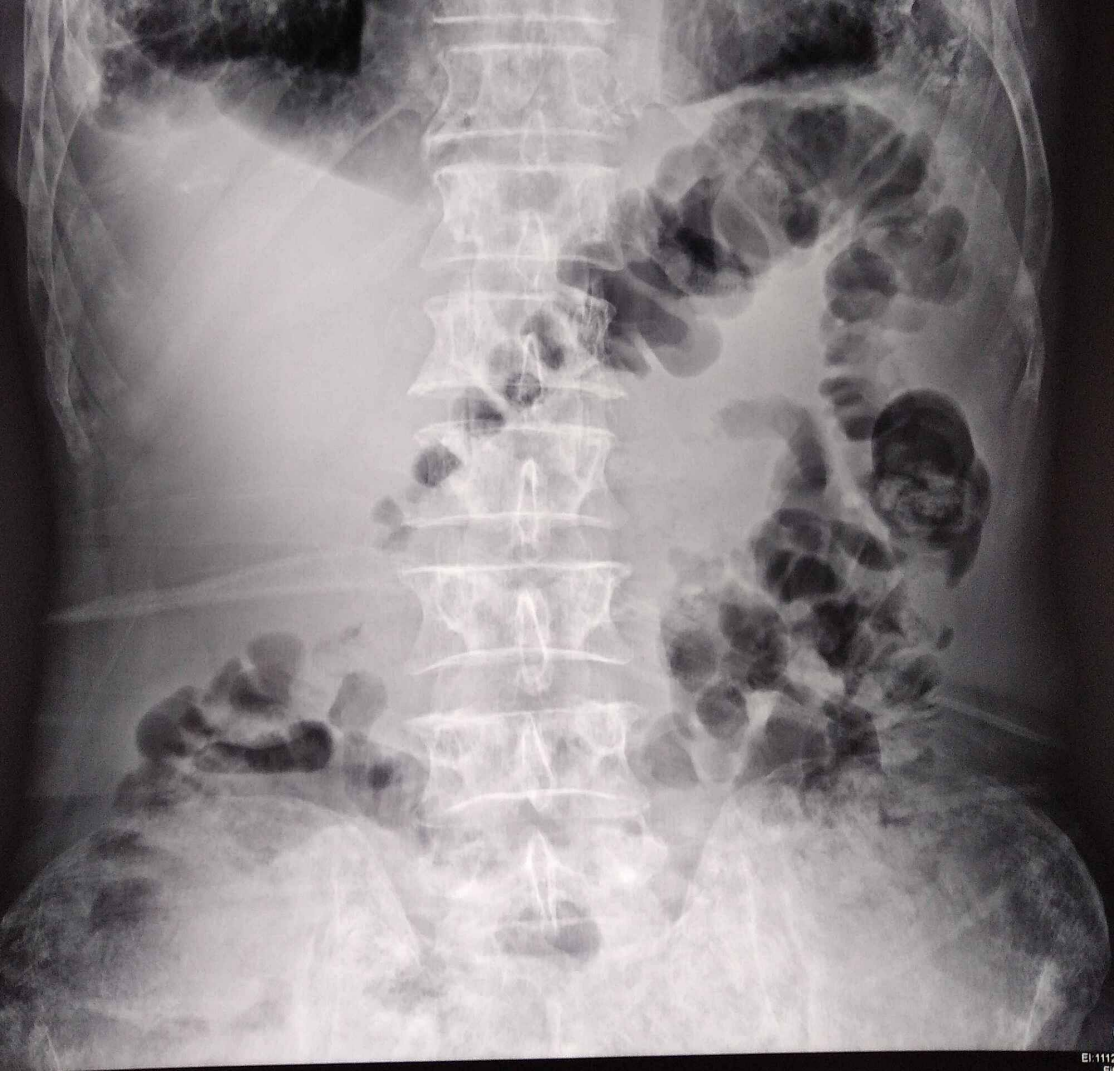
No significant finding on abdominal X-ray

**Figure 2:**
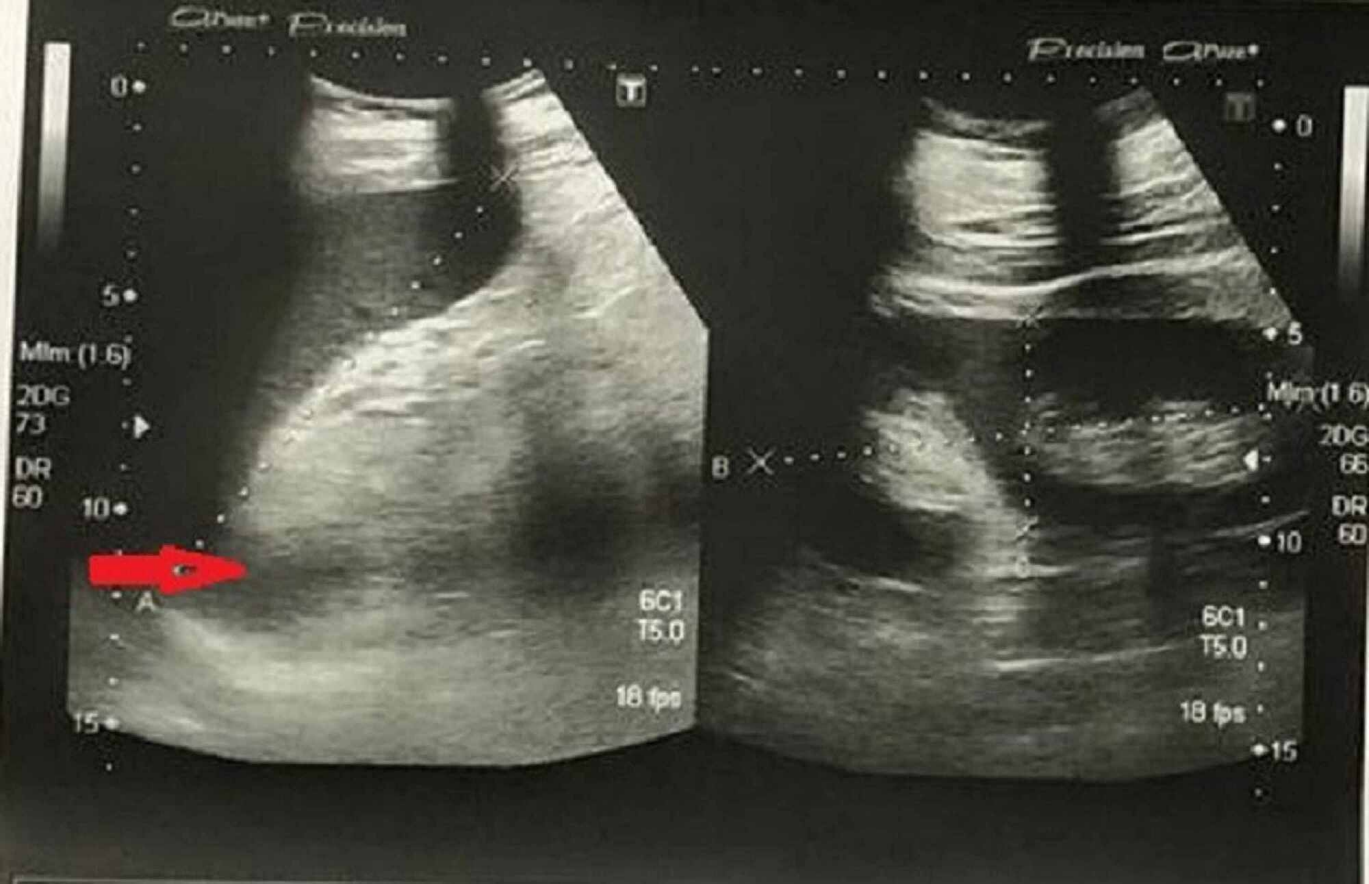
Ultrasound abdomen shows fluid levels (red arrow)

Abdominal X-ray was insignificant (Figure 1) and ultrasound showed multiple fluids levels (Figure 2).

Her condition deteriorated despite conservative management, and an emergency exploratory laparotomy was done, which revealed complete thrombosis of the superior mesenteric artery and its branches with gangrene of the whole of the small gut, except proximal 3 feet from the duodenojejunal junction and distal one and a half feet from the ileocecal junction. Resection of the gangrenous gut with exteriorization of both ends was done. On the basis of the above findings, a thrombophilia screen (Factor v Liden mutations, factor S and C deficiency, antithrombin 3 levels), and anti-neutrophilic antibodies (ANA) were sent, which came out negative (Table 2).

**Table 2:** Thrombophilia screen and ANA results ANA: anti-neutrophilic antibodies

Factors	Patient Value	Normal Ranges
Factor V Leiden Screening	1.03 ratio	0.8-1.1 ratio
Anti-thrombin 3 level	120%	75-125%
Protein C	139 %	69-140 %
Free protein S	90%	67-140%
ANA	Negative (titer <1:80)	Titer <1:80

An echocardiogram was done, which showed no thrombus and an ejection fraction of 52%. She was discharged on home medication on the tenth postoperative day and was advised to follow up in two weeks. She was doing well on follow-up visits.

## Discussion

SARS-CoV-2 doesn’t only target the lungs but also affects other organs of the body [3]. It has also been implicated in coagulopathy, leading to venous as well as arterial thrombosis, especially in patients with severe disease [5]. Although the exact underlying mechanism of thromboembolism associated with COVID-19 is unclear, Virchow’s triad is by far the most common, explainable mechanism of thromboembolism in COVID-19 patients. One of the contributing factors for thrombosis could be reduced venous flow due to prolonged bed rest in critically ill COVID-19 patients [6]. Likewise, SARS-CoV-2 can cause direct damage to the vessel wall and subsequent coagulopathy by binding to the ACE-2 receptors that are found abundantly on endothelial cells. Furthermore, some studies suggest that significantly elevated angiotensin 2 in COVID-19 patients leads to activation of renin-angiotensin 2 and widespread endothelial damage [7]. Another possible explanation for venous thromboembolism (VTE) in patients with COVID-19 is hypoxia because hypoxic conditions have been associated with an increased risk of thrombosis [8].

Our patient was diagnosed with acute mesenteric ischemia after contracting COVID-19 recently, and he was thoroughly investigated for the possible common causes of mesenteric ischemia in a young patient, such as autoimmune diseases, including vasculitis, atrial fibrillation, abdominal malignancies, and inflammatory bowel disease, but everything, including thrombophilic screening, was normal [9]. A causal relationship was established between COVID-19 and hypercoagulability in our patient after ruling out the major predisposing factors for thromboembolus formation.

Coagulopathy and increased D-dimers level in hospitalized patients with COVID-19 has been linked with a high mortality rate because it is quite challenging to manage these patients, as proper studies are lacking in this part [10]. However, as a general approach, every patient with COVID-19 should be given pharmacological thrombo-prophylaxis, most preferably intravenous unfractionated heparin or low molecular weight heparin (LMWH), unless contraindicated [10]. The dose can be adjusted to the patient’s weight and underlying condition. Our patient didn't receive any anticoagulant on the initial visit, as she was having mild COVID-19 symptoms. Later on, she ended with thrombotic complications.

## Conclusions

All COVID-19 patients should be followed regularly with coagulation profiles such as Prothrombin time (PT), activated partial thromboplastin time (APTT), international normalized ratio (INR), platelets, and D-dimer levels to monitor the progression of the disease, for timely intervention, and for adjusting the thrombolytic dosage. During this pandemic, physicians should keep in mind the possible thrombotic complications of COVID-19 because timely intervention can save the life of the patient.

## References

[1] Lai CC, Shih TP, Ko WC, Tang HJ, Hsueh PR (2020). Severe acute respiratory syndrome coronavirus 2 (SARS-CoV-2) and coronavirus disease-2019 (COVID- 19): the epidemic and the challenges. Int J Antimicrob Agents.

[2] (2020). World Health Organization. Coronavirus Disease 2019 (COVID-19) situation report - 202. https://www.who.int/docs/default-source/coronaviruse/situation-reports/20200809-covid-19-sitrep-202.pdf.

[3] Patel KP, Patel PA, Vunnam RR, Hewlett AT, Jain R, Jing R, Vunnama SR (2020). Gastrointestinal, hepatobiliary, and pancreatic manifestations of COVID-19. J Clin Virol.

[4] Wise J (2020). Covid-19 and thrombosis: what do we know about the risks and treatment?. BMJ.

[5] Helms J, Tacquard C, Severac F (2020). High risk of thrombosis in patients with severe SARS-CoV-2 infection: a multicenter prospective cohort study. Intensive Care Med.

[6] Phillippe HM (2017). Overview of venous thromboembolism. Am J Manag Care.

[7] Guo J, Huang Z, Lin L, Lv J (2020). Coronavirus disease 2019 (COVID-19) and cardiovascular disease: a viewpoint on the potential influence of angiotensin-converting enzyme inhibitors/angiotensin receptor blockers on onset and severity of severe acute respiratory syndrome coronavirus 2 infection. J Am Heart Assoc.

[8] Gupta N, Zhao YY, Evans CE (2019). The stimulation of thrombosis by hypoxia. Thromb Res.

[9] Ozturk G, Aydinli B, Atamanalp SS, Yildirgan MI, Ozoğul B, Kısaoğlu A (2012). Acute mesenteric ischemia in young adults. Wien Med Wochenschr.

[10] Tang N, Li D, Wang X, Sun Z (2020). Abnormal coagulation parameters are associated with poor prognosis in patients with novel coronavirus pneumonia. J Thromb Haemost.

